# Physical therapies as an adjunct to Botulinum toxin-A injection of the upper or lower limb in adults following neurological impairment

**DOI:** 10.1186/2046-4053-1-29

**Published:** 2012-06-26

**Authors:** Bianca Z Kinnear

**Affiliations:** 1 Occupational Therapist specializing in neurological rehabilitation in HammondCare. Level 2, 447 Kent St, Sydney, NSW, 2000, Australia

**Keywords:** Spasticity, Physical Therapy, Occupational Therapy, Botulinum toxin-A, Neurology, Motor Disorder

## Abstract

**Background:**

Spasticity of muscles is a common consequence of central nervous system impairment. Traditionally, neurological rehabilitation for spasticity has involved occupational and physical therapy; however, increasingly Botulinum toxin–A injections may be provided. Injection effects are temporary. Consequently, understanding the effect of adjunct physical therapies will help inform multimodal rehabilitation decisions. Presently, these effects are not known. This systematic review will identify and summarize evidence on physical therapies used after Botulinum toxin-A injection to improve motor function in adults with neurological impairments.

**Method:**

Systematic searching of seven electronic databases will occur to identify relevant randomized trials. Available trial data will be extracted into a list of pre-defined primary outcomes, including range of movement, spasticity and functional limb use. Pre-defined secondary outcomes will also be reviewed where trials have these data available for reporting. Effects will be expressed as mean differences or standardized mean differences with 95% confidence intervals (CI). Where possible, comparable results will be meta-analyzed, and a summary of the available pool of evidence produced.

All randomized controlled trials will be rated using the PEDro methodological quality scale. Where possible, study data will be meta-analyzed using RevMan 5 Software. The protocol was registered in PROSPERO international prosepective register of systematic reviews (PROSPERO 2011:CRD42011001491).

**Discussion:**

Review results will be the most comprehensive answer available to the following question: Are physical therapies clinically effective after Botulinum toxin-A injections in adults with neurological spasticity? Results will inform healthcare providers and managers who determine who gets access to and provision of Botulinum toxin-A injection and whether this is done with or without physical therapies. Results will inform the clinicians who conduct physical therapy following injection. This protocol provides readers with the scope and depth of a search that will ultimately answer a complex and pressing treatment question. The variability of current practice and high level of expense associated with multimodal rehabilitation means review results will be more useful and less contestable if the protocol is revealed in full through advance publication.

## Background

Spasticity is one negative symptom which can occur following central nervous system impairment [1,2]. Spasticity is defined as a velocity-dependent increase in stretch reflex with exaggerated tendon jerks [1]. This motor disorder can have a profound impact on health outcomes and quality of life. It is associated with pain [3], and often has a negative impact on function, self-esteem and body image [4]. General health can suffer as spasticity leads to difficulties in hygiene and maintaining skin integrity in affected limbs with consequent infection common [5]. Treatment of spasticity and management of consequences of this motor disorder is an unending, expensive and time intensive task for health services and the people affected by it, with results recognized to be ‘less than satisfactory’ [6]. There is an urgent need to identify treatment that is safe, effective and efficient [7]. In the past decade, traditional rehabilitation for this motor disorder, such as occupational and physical therapy, has been augmented by pharmacotherapy, such as Botulinum Toxin-A, which has been demonstrated to reduce spasticity [8]. The effect from pharmacotherapy is temporary so there is interest in the adjunct effects of physical therapies, since the rehabilitation goals of patients are long term. Typical goals include: increasing range of motion (ROM), improving limb position and performance of activities, and ensuring skin integrity [9-11].

Following BoNT-A injection, physical therapies can be used, such as stretching, casting, strengthening exercises, splinting and movement training often in the context of functional activities where normal movement patterns are encouraged [2,6,12,13]. These physical therapies post-BoNT-A injection are thought to improve ROM and function in patients with motor disorders following neurological impairment. Both BoNT-A and physical therapies have been shown, together and separately, to have an impact on health outcomes, such as walking ability [14,15]. To date, however, no study has examined the cumulative evidence regarding combined BoNT-A and physical therapies for treatment of spasticity in upper or lower limbs of adult patients with neurological impairment. Given the cost and scale of the service provided, and the breadth and complexity of health and quality of life problems involved, a review that provides a comprehensive summary of evidence relating to this problem and intervention is urgently needed.

## Methods

This systematic review will identify and summarize evidence on physical therapies used after Botulinum toxin-A injection to improve motor function in adults with neurological impairments. In summarizing evidence, the review will evaluate the effectiveness of physical therapies used post-BoNT-A injection for improving motor function in this patient population. Inclusion criteria for studies in the review now follow.

### Types of studies

Eligible studies will be randomizsed controlled trials and quasi-randomized controlled trials. Only the first arm of cross-over trials will be included.

### Types of participants

Eligible participants will be aged 16 years and over with a neurological impairment (central nervous system damage) and have received BoNT-A injections in their limb muscles up to three months prior to study rehabilitation commencement for spasticity management.

### Types of interventions

Physical rehabilitation interventions provided to patients who have received BoNT-A to the upper or lower limb, including casting, splinting, stretching, movement training, exercises, strengthening or electrical stimulation, are compared with sham or no physical therapy.

### Types of outcome measures

#### *Primary outcomes*

1. Measures of range of movement, such as goniometry or torque-controlled range of movement.

2. Measures of improved functional limb use or active movement of the affected limb as measured on the Action Research Arm Test, the Motor Activity Log (assessment or self report), Wolf Motor Function Test, Fugl-Meyer assessment of motor function, Box and Blocks test, velocity of gait (meters/second) and step length.

3. Measures of reduced spasticity as measured by the Modified Ashworth Scale.

#### *Secondary outcomes*

1. Health-related quality of life

2. Caregiver/attendant care time and/or number

3. Discharge destination/living situation

4. Rehabilitation length of stay

5. Mortality

6. Adverse events

Studies will be excluded if:

i) The study design investigated diagnostic or prognostic factors/relationships

ii) Less than 50% of the interventions were applied to the upper or lower limb

iii) Outcomes from physical therapies were not able to be differentiated from the outcomes of other therapies provided simultaneously to participants.

### Search strategy

The following databases will be electronically searched for all available years: PEDro, CENTRAL, Pub Med, EMBASE, CINAHL, National Research Register, Meta Registry of Controlled Trials and Occupational Therapy Systematic Evaluation of Effectiveness database (OTseeker). The search will not be limited by date or publication status. We will check the reference lists of any eligible studies identified for further relevant studies. Unpublished, non-peer reviewed sources, such as conference abstracts, will not be included (refer to appendix 1.)

Two authors will independently review all potential studies for inclusion against the eligibility criteria. They will examine the title and abstract and, where necessary, the full text of studies to assess if they are eligible for inclusion. If they cannot reach agreement by discussion, a third author will make the final decision about eligibility.

### Data extraction

Two authors will independently use a standard form to extract study characteristics and outcome data from the studies. Discrepancies will be checked against the original data. A third author will make the final decision if there is a disagreement. One author (BK) will enter data in Revman meta-analysis software (Review Manager (RevMan) [Computer program]. Version 5.1. Copenhagen: The Nordic Cochrane Centre, The Cochrane Collaboration, 2011) [16]. Data will be reported *during* therapy as well as *at end* of therapy. Any outcomes measured after therapy has finished will be grouped as less than one month, one to six months, and over six months.

### Quality assessment

Methodological quality will be assessed using the PEDro scale by one reviewer (BK). The PEDro scale has established reliability and provides a score out of 10 [17]. Studies which attain a PEDro score of 7 or greater are considered ‘high quality’, those with a PEDro score of 5 or 6 are considered ‘moderate quality’ and those with a PEDro score of 4 or less are considered ‘poor quality’ in terms of study methods and susceptibility to bias [18]. Adequacy of concealment will additionally be rated using the procedure outlined by Schulz [19]. The methodological quality of included observational studies will be assessed independently by two raters using the MOOSE (Meta-analysis Of Observational Studies in Epidemiology) guidelines [20]. Scores will be based on all information available from both the published version and the authors themselves. No trial will be excluded on the basis of poor quality.

### Data analysis

Analysis of covariance-adjusted between-group means and standard deviations will be extracted in preference to between-group differences in change scores, and between-group differences in change scores will be extracted in preference to between group differences in final scores. If studies reported data as medians and interquartile ranges, medians will be used as a surrogate for means and standard deviations were estimated as 80% of the interquartile range (studies which do not report an interquartile range (IQR) will be excluded from the meta-analysis). For continuous outcome measures, a pooled estimate of treatment effect will be determined by calculating the mean difference and the corresponding 95% CIs. For dichotomous outcome measures, a pooled estimate of treatment effect will be calculated for each outcome across studies using risk ratio where appropriate and the corresponding 95% confidence intervals (CIs). Time-to-event data will be analyzed using the hazard ratio and 95% CIs as required. When conducting a meta-analysis combining results from crossover studies, the first-arm data only will be used. In the event of missing, incomplete or unclear data, the original investigators will be contacted. If it is not possible to obtain the necessary data for analysis, the study results will be described in the text and will not be included in the meta-analysis.

When there are at least two clinically homogenous studies (studies which investigated the effect of similar interventions on similar populations and reported similar outcomes) meta-analysis will be considered. In such circumstances, the I^2^ statistic will be used to quantify the heterogeneity of outcomes and informed decisions about whether to pool data. Where I^2^ is greater than 30% in the presence of significant chi-squared test result (*P*-value < 0.10), this will be interpreted as indicating heterogeneity [21]. If there is significant heterogeneity (over 50%), data will not be pooled.

We will enter data extracted from included studies into RevMan software [16]. Meta-analyses of clinically homogenous therapies will be conducted using a fixed-effects model. Where heterogeneity is substantial (I2 > 50%), the possible causes of heterogeneity will be explored in sensitivity analyses in which individual studies will be omitted one at a time. Should heterogeneity not be able to be explained, a random-effects model will be used.

### Sensitivity analysis

The robustness of our results will be tested through sensitivity analyses excluding unpublished studies, small studies and studies with a PEDro score less than 5.

## Discussion

Spasticity can be a significant causal factor in patients’ decreasing functional limb use and mobility, often compounding the difficulties faced in rehabilitation by patients with motor weakness and impairment. Reviewing the effectiveness of therapies provided after BoNT-A and the effects on spasticity, movement and function will provide much needed guidance to therapists, as well as policy and funding guidance to health departments in how to provide the most effective interventions.

There are currently no evidence-based guidelines for the physical rehabilitation that should be provided to adults with neurological impairment who have received Botulinum toxin–A injections in the upper or lower limb. Recent European international consensus guidelines [22] and national treatment guidelines [23] do not recommend a protocol for physical rehabilitation when provided as an adjunct to Botulinum toxin injection. Instead, evidence is available from completed and ongoing trials in the area of stroke rehabilitation where different post Botulinum toxin injection physical rehabilitation interventions are compared with each other, other forms of therapy or no therapy. Internationally, Botulinum toxin-A has regulatory approval for upper and lower limb injection in adults with neurological conditions. This review considers whether physical rehabilitation following such injection has an effect on functional outcomes.

## Appendix 1

### A.1. PEDro Search Strategy

1. Spasticity

2. Botulinum toxin

### A.2. CENTRAL Search Strategy

1. Spasticity (key word, title, abstract)

2. Botulinum toxin (key word, title, abstract)

3. Therapy (key word, title, abstract)

4. Adults (key word, title, abstract)

### A.3. PubMed Search Strategy

1. Muscle spasticity/

2. Spastic.tw

3. Muscle tonus

4. Hyperton$

5. ((muscle* or muscular) adj3 (spasm* or cramp or spastic* or clonus).ab, ti.

6. or/1–5

7. Stroke/

8. Cerebrovascular disorders

9. exp brain ischemia

10. brain/or exp intracranial arterial diseases

11. exp “intracranial embolism and thrombosis”

12. exp intracranial haemorrhages/

13. ((stroke$ or post stroke$ or post- stroke$ or cva$).tw.)

14. ((cerebrovascular cerebral vascular) adj3 (accident? or insult?).tw.)

15. (((cerebral or brain$ or vertebrobasilar) adj5 (infarc$ or isch?emi$ or thrombo$ or apoplexy or emboli$)).t.w.)

16. ((cerebral or brain or subarachnoid) adj5 (haemorrhage or hemorrhage or haematoma or hematoma or bleed)).tw.)

17. (((trauma$ or acquired) adj5 brain injur$).tw.)

18. (exp brain injuries/)

19. brain damage, chronic/or brain injury, chronic/)

20. (exp craniocerebral trauma/or head injuries, closed/or intracranial hemorrhage, traumatic/)

21. (exp encephalitis/or exp meningitis, viral/or brain abscess/or exp central nervous system infections)

22. ((encephalitis or meningitis or brain abscess or brain infection$ or cerebral infection$).tw.)

23. (exp Brain Neoplasms/)

24. (((brain or cerebr$) adj5 (neoplasm$ or lesion$ or tumor$)).tw.)

25. Brain injuries/

26. Traumatic brain injury*

27. Spinal injuries/

28. exp Spinal cord injuries

29. (spinal cord adj3 (injur* or damage* or contusion* or lacerat* or trauma*)).ab,ti.

30. 28 or 29 or 30

31. Cerebral palsy or CP

32. cerebral pals$.tw

33. Multiple sclerosis or MS

34. ((((“Multiple Sclerosis”[mh]) OR (“Myelitis, Transverse”[mh:noexp]) OR (“Demyelinating Diseases”[mh:noexp]) OR (“Encephalomyelitis, Acute Disseminated”[mh:noexp]) OR (“Optic Neuritis”[mh])) OR (((“multiple sclerosis”) OR (“neuromyelitis optica”) OR (“transverse myelitis”) OR (encephalomyelitis) OR (devic) OR (“optic neuritis”)) OR (“demyelinating disease*”) OR (“acute disseminated encephalomyelitis”)))

35. Hemiplegia/

36. Hemiplegi$.tw

37. Monoplegi$.tw

38. Triplegi$.tw

39. Quadriplegia/

40. Quadriplegi$.tw

41. or/7–40

42. exp Botulinum Toxins/

43. Botulinum toxin type a/

44. Botulin$.tw

45. Botox.tx

46. Dysport. tw

47. Muscle spasticity/dt [Drug therapy]

48. Muscle tonus/de [Drug effects]

49. or/42–48

50. physical therapy.tw

51. Physical therapy modalities/

52. Exercise therapy/

53. Occupational therapy/

54. Exercise movement techniques/

55. Rehabilitation/

56. Splints/

57. Casts, Surgical/

58. Treatment outcome/

59. Orthotic devices/

60. or/46–55

61. clinical trial.pt

62. controlled clinical trial.pt.

63. clinical trials as topic.sh.

64. trial.ti.

65. randomi?ed.ab.

66. randomized controlled trial.pt

67. randomly.a.b.

68. or/57–63

69. humans.sh

70. 6 and 41 and 49 and 60 and 68 and 69

### A.4. CINAHL Search Strategy

1. Muscle spasticity

2. Muscle tonus

3. Muscle hypertonia

4. Muscle cramp

5. Muscles

6. Or/1–5

7. Stroke

8. Cerebrovascular disorders

9. Cerebral ischemia

10. Brain

11. Intracranial arterial diseases

12. Intracranial embolism and thrombosis

13. Cerebral aneurysm

14. Hemorrhage

15. Subarachnoid hemorrhage

16. Infarction

17. Hypoxia, brain

18. Brain death

19. Brain damage, chronic

20. Hypoxia-Ischemia, Brain

21. Encephalitis

22. Meningitis, viral

23. Brain abscess

24. Brain neoplasms

25. Brain injuries

26. Spinal injuries

27. Spinal cord injuries

28. Spinal cord neoplasms

29. Cerebral palsy

30. Multiple sclerosis

31. Myelitis

32. Encephalomyelitis, acute disseminated

33. Demyelinating autoimmune diseases, CNS

34. Hemiplegia

35. Quadriplegia

36. Or/7–35

37. Botulinum Toxins

38. Botulism

39. Drug Therapy

40. or/37–39

41. Physical therapy

42. Physical therapy assessment

43. Physical therapy service

44. Therapeutic exercise

45. Occupational Therapy

46. Occupational Therapy assessment

47. Rehabilitation

48. Splints

49. Casts

50. Treatment outcomes

51. Orthoses

52. Or/41–51

53. Human

54. Adult

55. 53 or 54

56. 6 and 36 and 40 and 52 and 55

## Competing interests

The author had an untied educational grant of $3,000 from Allergan Australia Pty Ltd to support her systematic review methodology skill development at independently conducted workshops. She has no other financial relationship with any organization that might have an interest in the submitted work in the previous three years. The study is being conducted as part of the author’s PhD program, under supervision of independently funded senior academics at the University of Wollongong (Anne Cusick) and at La Trobe University (Natasha Lannin).
